# Langerhans Cell Histiocytosis of the Clavicle in a 13-Year-Old Boy

**DOI:** 10.1155/2014/510287

**Published:** 2014-01-05

**Authors:** Shital N. Parikh, Vishal R. Desai, Anita Gupta, Christopher G. Anton

**Affiliations:** ^1^Division of Pediatric Orthopaedic Surgery, Cincinnati Children's Hospital Medical Center, MLC 2017, 3333 Burnet Avenue, Cincinnati, OH 45229, USA; ^2^Singapore General Hospital, Outram Road, Singapore 169608; ^3^Division of Pathology, Cincinnati Children's Hospital Medical Center, MLC 1035, 3333 Burnet Avenue, Cincinnati, OH 45229, USA; ^4^Division of Radiology, Cincinnati Children's Hospital Medical Center, MLC 5031, 3333 Burnet Avenue, Cincinnati, OH 45229, USA

## Abstract

Langerhans Cell Histiocytosis (LCH) is a rare neoplasm characterized by abnormal proliferation of histiocytic cells. In this case report, we describe a unique case of a 13-year-old boy who presented to the clinic with an insidious onset of mid-clavicular pain. The provisional radiologic diagnosis of Langerhans Cell Histiocytosis of the clavicle was confirmed by an incisional biopsy of the left mid-clavicle lesion. The patient's lesion was treated by curettage, bone grafting, and internal fixation, due to the presence of pathologic fracture. At the 2-year followup, the patient was asymptomatic, and the lesion showed complete resolution without recurrence. The case report highlights the characteristic features of Langerhans Cell Histiocytosis in an unusual location, the knowledge of which would help avoid delayed or missed diagnosis in the future.

## 1. Introduction

Langerhans Cell Histiocytosis (LCH) is the current nomenclature for disorders previously known as histiocytosis X, which grouped eosinophilic granuloma, Hand-Schuller-Christian disease, and Letterer-Siwe disease [1–4]. LCH is an abnormal proliferation of tissue macrophages called Langerhans cells in one or more organs, including bone, skin, lymph nodes, lung, liver, spleen and bone marrow [1–4]. Patient age typically ranges from 5 to 15 years in about 90% of the cases, with a slight male predominance [1, 2]. The estimated incidence of LCH is 0.2–0.5 per 100,000 children per year in the USA. Majority (79%) of LCH are solitary lesions. The most frequent site of occurrence of LCH is the skull, followed by femur, jaw, pelvis, ribs, spine, scapula, humerus, and sternum [2]. Though rare, LCH of clavicle has been reported [5–8].

## 2. Case Presentation

A 13-year-old Caucasian male presented to clinic with an insidious onset of nondominant, left-sided, mid-clavicular pain for six weeks of duration. The mother described the texture of this area as “sand-like.” There was no history of trauma, fever, chills, night sweats, weight loss, joint pain, or swelling. On physical examination, the left clavicle demonstrated an isolated, tender, firm, nonerythematous, nonfluctuating, soft tissue swelling directly over the mid-clavicle without a noticeable deformity. Skin changes were absent. Laboratory examination showed total blood count of 8.9 K/UL (4.5–13.5) with normal differential. Erythrocyte sedimentation rate (ESR) was 14 mm/hour (0–10) and C-reactive protein (CRP) was 1.3 mg/dL (<1.0).

Conventional radiograph of the left clavicle and a Tc-99m-MDP bone scan were performed. Subsequently CT scan and MRI were performed. An incisional biopsy of left mid-clavicle was performed in the operating room and the fragments of soft red-tan tissue were sent for histopathologic examination. It showed a histiocytic neoplasm admixed with eosinophils, confirming the diagnosis of Langerhans Cell Histiocytosis (LCH). Cultures were negative. The lesion was treated by curettage, bone grafting, and internal fixation with a 3.5 mm reconstruction plate due to the presence of pathologic fracture. At 2 years of followup, patient was asymptomatic and the lesion showed complete resolution without recurrence.

## 3. Discussion

### 3.1. Imaging

**Figure 1:**
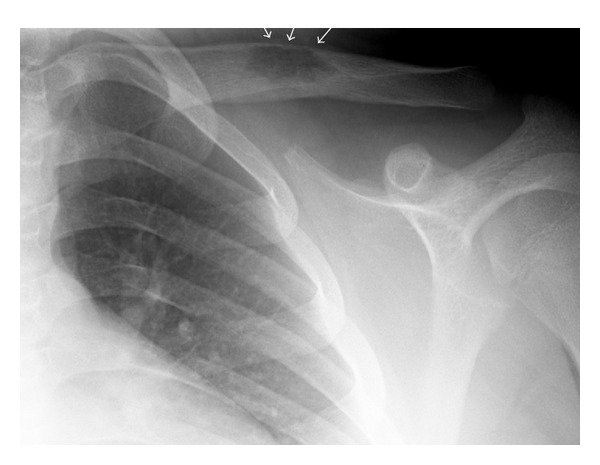
An AP radiograph of the left clavicle in a 13-year-old boy shows a mid-clavicular lytic lesion and subtle periosteal reaction superiorly (arrows). No internal matrix, surrounding sclerosis or soft tissue mass is seen.

**Figure 2:**
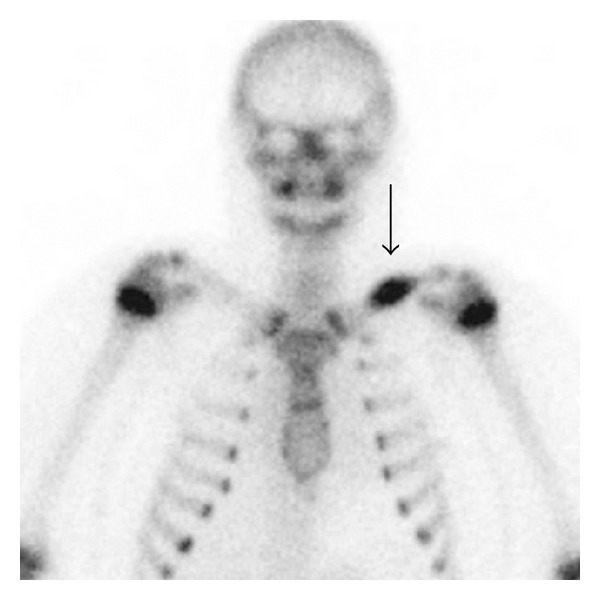
A Tc-99m-MDP bone scan AP image shows intense focus of radioisotope accumulation within the mid-left clavicle (arrow). Incidental radioisotope uptake from dental disease and normal increased uptake in bilateral proximal humerus physis in a skeletally immature patient is seen.

**Figure 3:**
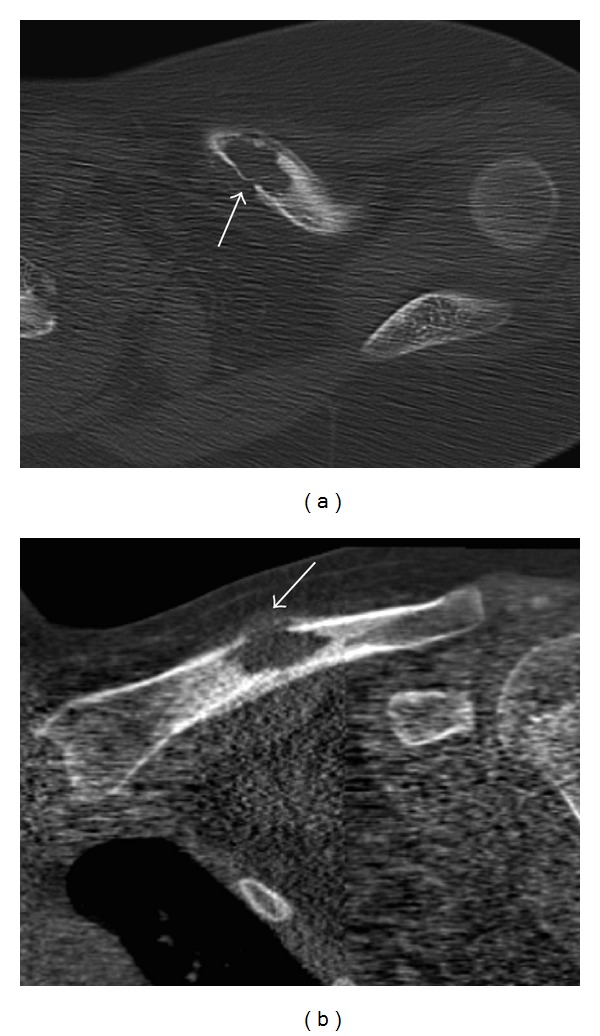
Axial (a) and coronal (b) reformatted CT images in bone window show the lytic mid-clavicle lesion with periosteal reaction and irregular cortical disruption (arrow). No soft tissue mass or marginal sclerosis is detected.

**Figure 4:**
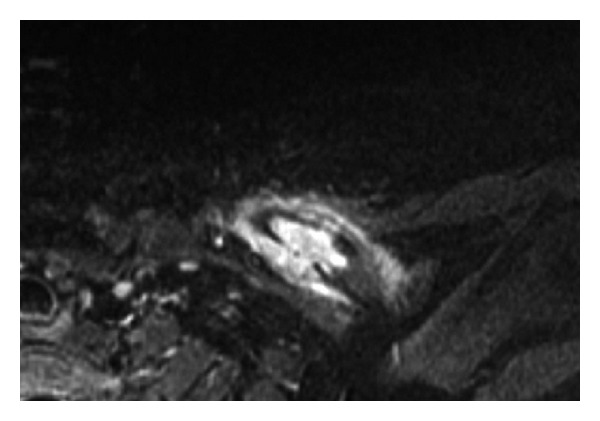
An axial STIR MR image shows a hyperintense mid-left clavicle lesion with periosteal reaction anteriorly. Moderate surrounding soft tissue hyperintense signal or edema is detected without discrete soft tissue mass or neurovascular invasion. The posterior cortical disruption is noted.

The radiographic appearance of typical solitary LCH within a long bone is a lytic, medullary based, metaphyseal, or diaphyseal lesion, with or without periosteal reaction (Figure 1). The periosteal reaction depends on cortical erosion [1–3]. There is lack of marginal sclerosis. Increased uptake of the radiopharmaceutical is seen on bone scan, which is performed to detect other sites of disease (Figure 2). CT scan (Figure 3) and MRI (Figure 4) are useful modalities in evaluating soft tissue involvement, which is normally absent or minimal in LCH [1].

### 3.2. Differential Diagnosis

The most common causes of nontraumatic lesions of clavicle are neoplasm, infection, or developmental abnormalities [5]. Majority of the neoplasms occurring in clavicle are malignant. Kapoor and Tiwari reported 8 primary malignant tumors of the clavicle, including four Ewing sarcomas in their series of 12 patients with clavicle tumors [6]. Ewing sarcoma is the most common sarcoma to occur in flat bones (pelvis, ribs, and face) and usually is seen in the first or second decade of life. Radiographic features typical of a Ewing sarcoma include an ill-defined, permeative, destructive lesion with aggressive periosteal reaction and soft tissue mass. MRI findings of soft tissue involvement or areas of localized hemorrhagic necrosis of lesion are characteristic features of Ewing sarcoma [6]. Osteomyelitis can present as a tender, firm swelling on examination, and as an osteolytic lesion with periosteal reaction on imaging studies [6, 9]. Laboratory values including ESR and CRP are typically elevated in osteomyelitis. Besides Ewing sarcoma and osteomyelitis, other differential diagnoses of the lesion like giant cell tumor of bone and nonossifying fibroma are less likely.

### 3.3. Management

**Figure 5:**
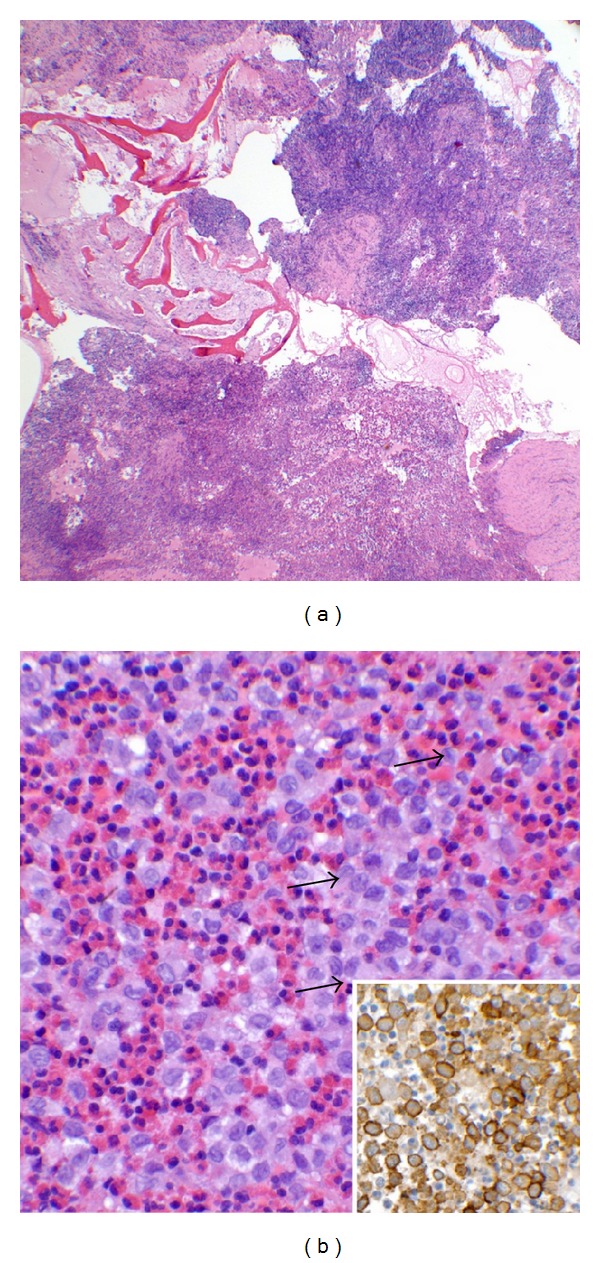
(a) Low magnification shows tumor cells expanding and destroying bony trabeculae (hematoxylin-eosin stain, original magnification ×40). (b) High magnification demonstrates tumor cells characterized by coffee bean-shaped grooved nuclei indicated by the arrow, admixed with eosinophils consistent with Langerhans cells (hematoxylin-eosin, original magnification ×400). Insert: CD1a immunostain is a transmembrane antigen normally found on Langerhasn cells (CD1a immunostain, original magnification ×600).

Biopsy is performed to confirm the diagnosis of LCH. On histology, sections show a histiocytic neoplasm admixed with eosinophils expanding and destroying bone (Figure 5(a)). The tumor cells are large and polygonal with eosinophilic cytoplasm, coffee bean-shaped nuclei with occasional longitudinal grooves, vesicular nuclear chromatin, and inconspicuous nucleoli consistent with Langerhans cell histiocytes (Figure 5(b)). These neoplastic cells are immunoreactive to CD1a (Figure 5(b) insert). Cultures are taken at the time of biopsy. Treatment of LCH depends on the extent of the disease. Various forms of treatment for a solitary lytic lesion affecting a long bone have been attempted. The therapeutic modalities include observation for spontaneous resolution, biopsy, curettage with or without bone grafting, local steroid injection, anti-inflammatory drugs, bisphosphonates, radiotherapy, chemotherapy, and immunotherapy [4]. The result of treatment for solitary LCH lesion is more satisfactory than the treatment for multifocal or systemic disease, though recurrence has been reported [4].
